# Transfer of malignant trait to *BRCA1* deficient human fibroblasts following exposure to serum of cancer patients

**DOI:** 10.1186/s13046-016-0360-9

**Published:** 2016-05-14

**Authors:** Dana Hamam, Mohamed Abdouh, Zu-Hua Gao, Vincenzo Arena, Manuel Arena, Goffredo Orazio Arena

**Affiliations:** Cancer Research Program, McGill University Health Centre-Research Institute, 1001 Decarie Boulevard, Montreal, H4A 3J1 QC Canada; Department of Experimental Surgery, Faculty of Medicine, McGill University, 845 Rue Sherbrooke O, Montreal, H3A 0G4 QC Canada; Department of Pathology, McGill University Health Centre-Research Institute, 1001 Decarie Boulevard, H4A 3J1 Montreal, QC Canada; Department of Obstetrics and Gynecology, Santo Bambino Hospital, via Torre del Vescovo 4, Catania, Italy; Department of Surgical Sciences, Organ Transplantation and Advances Technologies, University of Catania, via Santa Sofia 84, Catania, Italy; Department of Surgery, McGill University, St. Mary Hospital, 3830 Lacombe Avenue, Montreal, H3T 1M5 QC Canada

**Keywords:** Metastasis, Genometastasis, Fibroblasts, Tumor suppressor genes, BRCA1, Transformation, Exosomes

## Abstract

**Background:**

It was reported that metastases might occur via transfer of biologically active blood circulating molecules from the primary tumor to distant organs rather than only migration of cancer cells. We showed in an earlier study that exposure of immortalized human embryonic kidney cells (HEK 293) to cancer patient sera, induce their transformation into undifferentiated cancers due to a horizontal transfer of malignant traits. In the present work, we tested the hypothesis that even other human cells as long as they are deficient for a single oncosuppressor gene might undergo malignant transformation when exposed to human cancer serum.

**Methods:**

We used the CRISPR/Cas9 system to establish a stable *BRCA1* knockout (KO) in human fibroblasts. The *BRCA1*-KO fibroblasts were exposed to cancer patients’ sera or healthy patients’ sera for 2 weeks. Treated cells were analyzed for cell proliferation and transformation to study their susceptibility to the oncogenic potential of cancer patients’ sera and to determine the possible mechanisms underlying their hypothesized transformation.

**Results:**

*BRCA1*-KO fibroblasts treated with cancer patients’ sera displayed higher proliferation and underwent malignant transformation as opposed to wild type control fibroblasts, which were not affected by exposure to cancer patients’ sera. The malignant transformation was not seen when *BRCA1*-KO fibroblasts were treated with healthy human sera. Histological analysis of tumors generated by *BRCA1*-KO fibroblasts showed that they were carcinomas with phenotypical characteristics related to the cancers of the blood donor patients. Interestingly, *BRCA1*-KO fibroblasts were significantly more prone to internalize serum-derived exosomes, when compared to wild type fibroblasts. This suggests that oncosuppressor genes might protect the integrity of the cell genome also by blocking integration of cancer-derived exosomes.

**Conclusion:**

These data support the hypothesis that any human cells carrying a single oncosuppressor mutation is capable of integrating cancer factors carried in the blood and undergo complete malignant transformation. Oncosuppressor genes might protect the cell genome by impeding the integration inside the cells of these mutating factors.

**Electronic supplementary material:**

The online version of this article (doi:10.1186/s13046-016-0360-9) contains supplementary material, which is available to authorized users.

## Background

Metastasis is considered the leading cause of morbidity and mortality related to cancer [1, 2]. It has been well accepted that metastases develop by dissemination of cancer cells, which detach from the primary tumors, travel through the circulatory system and reach the metastatic site where they start to grow [3]. A review of recent literature brought evidence that the metastatic process might not be only due to primary tumor cells spreading to distant metastatic sites. Several studies reported that cancer cells-derived factors could either prepare a niche to permit the engraftment of malignant cancer cell in distant organs or predispose target cells, located in distant organs, to their malignant transformation [4–9]. These factors (i.e., proteins, nucleic acids, cell-surface receptors and lipids) could be either blood floating naked entities or molecules carried as cargo in exosomes [5, 7, 10–16].

Exosomes are small (30–100 nm) extracellular membrane-enclosed vesicles, which originate from cellular endosomal compartment under both physiological and pathological conditions [17–19]. Exosomes, which express distinctive surface markers, harbour substances that mirror the content of their cell of origin [20–22] and have the capability to induce different types effects, even at distance, by insuring the trafficking of different factors (i.e., survival and mitogenic signalling molecules) into target recipient cells [4, 8].

Pioneer researchers describing malignant trait transfer via blood circulating factors in immortalized mouse fibroblasts (NIH3T3 cells) called this phenomenon “genometastasis” [23–25]. Recently, after exposing immortalized human embryonic kidney cells (HEK293) to cancer patients’ sera, we observed their transformation into malignant cells, confirming for the first time the validity of the genometastatic theory in human cells [26]. In our study we remarked that only HEK293 were prone to undergo malignant transformation as opposed to different types of normal cells (fibroblasts, mesenchymal stem cells and embryonic stem cells), which failed to acquire the malignant traits.

Our findings supported the hypothesis that the different stages of carcinogenesis such as initiation, promotion and progression might not represent events limited to the cells forming the primary tumor, but may actually be a process reproducible in primed cells, located in target organs, through the incorporation of key factors released by the primary tumor. To strengthen our hypothesis that any cell with a single oncosuppressor mutation might be susceptible to integrate mutating factors at metastatic sites, we generated a human fibroblast cell line deficient for the oncosuppressor *BRCA1* (Breast cancer susceptibility gene 1) using the CRISPR technology and we exposed it to different types of patients’ cancer sera and healthy patients’ sera. *BRCA1* is a tumor suppressor gene that plays a significant role in DNA repair pathways [27]. Specific inherited mutations in *BRCA1* increase the risk of breast and ovarian cancers, and it has been associated with increased risks of several additional types of cancer [28–30].

The aims of our investigations were to determine the oncogenic potential of cancer patients’ sera on *BRCA1*-KO human fibroblasts, to characterize their differentiation following serum treatments and evaluate their phenotypes, and to determine their receptiveness to integrate serum-carried factors, such as exosomes. *BRCA1*-KO fibroblasts treated with cancer patients’ sera displayed higher proliferation and underwent malignant transformation as opposed to wild type fibroblasts, which were not affected by exposure to cancer patients’ sera. The malignant transformation was not seen when *BRCA1*-KO fibroblasts were treated with healthy human sera. Histological analysis of tumors generated by *BRCA1*-KO fibroblasts showed that they were carcinomas with phenotypical characteristics related to the cancers of the blood donor patients. Uptake of exosomes was significantly higher in the oncosuppressor mutated cells.

## Methods

### Patients’ recruitment and characteristics of cancers

Patients for the current study were recruited form the department of General Surgery at the Royal Victoria Hospital and St-Mary’s Hospital (Montreal, Canada) and underwent a written consent for blood collection in accordance to a protocol approved by the Ethics Committee of our institution (SDR-10-057). Blood samples were collected from both healthy individuals and patients who underwent resection of primary cancer and who were readmitted for metastatic disease treatment (Table 1). Healthy subjects were recruited based on three criteria: (i) age (35–45-year-old), (ii) absence of any signs and symptoms or personal history of cancer and (iii) negative family history for malignancy.

**Table 1:** Clinical features of cancer patients recruited in the present study

Cases	Age (y)	Gender	Tumor
A	70	Female	Adrenal corticocarcinoma - Lung Metastasis
B	69	Female	Breast cancer - Lung & Liver Metastasis
C	60	Female	Metastatic poorly differentiated neuroendocrine carcinoma
D	45	Female	Breast cancer - Liver Metastasis
E	66	Male	Colorectal cancer - Liver Metastasis
F	66	Male	Anal squamous cell carcinoma – Liver Metastasis
G	65	Male	Colorectal cancer - Liver Metastasis
H	72	Female	Colorectal cancer - Liver Metastasis
I	64	Female	Colorectal cancer - Liver Metastasis
J	73	Male	Pancreatic cancer

### Blood collection and serum preparation from cancer patients and healthy subjects

Blood samples (20 ml) were collected from a peripheral vein in vacutainer tubes (Becton Dickinson) containing clot-activation additive and a barrier gel to isolate serum. Blood samples were incubated for 60 min at room temperature to allow clotting and subsequently were centrifuged at 1500 g for 15 min. Serum was collected and a second centrifugation was performed on the serum at 2000 g for 10 min to clear it from any contaminating cells. Serum samples were aliquoted and stored at −80 °C until use.

### Cell line and culture conditions

Human fibroblasts and human embryonic kidney cells (HEK-293) (ATCC, VA, USA) were maintained as per supplier’s recommendations. When cells reached 30 % confluence, they were treated with DMEM-F12 medium (Wisent, Saint-Bruno, Canada) supplemented with antibiotics and 10 % cancer patient sera or control sera, which had been filtered through 0.2 μm filters. Cells were maintained in these conditions at 37 °C in humidified atmosphere containing 95 % air and 5 % CO_2_ with medium change every second day for 2 weeks. When cells reached 80-90 % confluence, they were passaged 1 in 6 using 0.05 % Trypsin-EDTA (Wisent, Saint-Bruno, Canada). To confirm that there was no contamination or carry-over of cells from human serum, aliquots of the culture medium were placed in a culture plate and incubated at 37 °C, 5 % CO2 for 4 weeks.

### CRISPR/Cas9-mediated *BRCA1* knockout in fibroblasts and cell sorting

We used the CRISPR/Cas9 system to establish a stable *BRCA1* knockout in human fibroblasts as previously described [31]. The pSpCas9(BB)-2A-GFP plasmid (PX458; Addgene, MA, USA) was used as the cloning backbone for sgBRCA1 (single-guided RNA to *BRCA1*). For this study, we designed two sequences targeting *BRCA1* locus (Table 2). Human fibroblasts were transfected with the empty plasmid (PX458) or the plasmid containing the guide (PX458-sgBRCA1) using Lipofectamine 3000 as per the manufacturer protocol (Invitrogen, Burlington, Canada). Transfected fibroblasts were then sorted based on the expression of the reporter GFP (green fluorescent protein) gene using a FACSAria cytometer (BD Biosciences, Mississauga, Canada) (Additional file 1: Figure S1). Sorted GFP positive cells were cultured and aliquots were subjected to Surveyor assay and Western blot analyses (Additional file 2: Figure S2). To minimize the off-target effects, cells were transfected with minimal amount of plasmid (500 ng). Also, the guide sequences were designed by using a web-based prediction algorithm tool [31]. We chose the highly ranked guide sequence with the least exonic off-target sites (Additional file 3: Table S2 and S3).

**Table 2:** Primers sequences used in sgBRCA1 cloning and knockout validation

Primer	Sequence (5’-3’)	Purpose
sgBRCA1^a^	CACCGTGGTCACACTTTGTGGAGAC AAACGTCTCCACAAAGTGTGACCAC	Single-guide cloning
hU6_Seq	ACTATCATATGCTTACCGTAAC	Primer for sequencing
BRCA1-F	TAGGGGTGGATATGGGTGAA	Surveyor assay
BRCA1-R	GTTGCAGTGAGCCAAGATCA	Surveyor assay

^a^These oligos were annealed to make the double-stranded linker with sticky ends (underlined nucleotides)

### SURVEYOR nuclease assay

DNA was isolated from sorted fibroblasts using GenElute Mammalian Genomic DNA Miniprep kit according to manufacturer specifications (Sigma, Oakville, Canada). Extracted DNA was amplified by PCR using Phusion High-Fidelity PCR kit (NEB, MA, USA) and set of primers for *BRCA1* gene (Table 2). The PCR reaction was performed in thermal cycler (Bio-Rad Laboratories, Inc., Hercules, CA, U.S.A.). Amplicons were loaded on 1 % agarose gel and corresponding bands with the expected sizes were excised and purified using QIAquick Gel Extraction Kit (QIAGEN, Redwood City, CA, USA). Purified DNA samples were subjected to the Indel (insertion/deletion) assay. Briefly, DNA was denatured at 95 °C for 10 min, and let anneal at decreasing temperatures (95 °C to 20 °C) for 30 min. Reannealed DNA was subjected to endonuclease digestion using IDT Surveyor Mutation Detection kit (IDT, Iowa, USA). The digestion products were run on 1 % agarose gel to quantify the Indel efficiency (Additional file 2: Figure S2) [31]. Briefly, the gel was imaged and the intensity of the bands in each lane was measured by using ImageJ Software. For each lane, we calculated the fraction of the PCR product cleaved by using the following formula: *fcut =* (*B + C*)/(*A + B + C*), where *A* is the intensity of the undigested PCR product, while *B* and *C* are the intensities of each cleaved band. The Indel percentage was estimated by applying the following formula:

$$ \mathrm{Indel}\left(\%\right)=100\times \left(1-\sqrt{\left(1-f\kern0.5em  cut\right)}\right) $$

### Population doubling level (PDL) calculation

Cells were considered at population doubling zero at the first time they are exposed to patient serum-containing culture medium. At every passage, cell number was determined and population doubling was calculated using the following formula; PDL = log(Nh/Ni)/log2, where Nh is the number of cells harvested at the end of the incubation time and Ni is the number of cells inoculated at the beginning of the incubation time. Cumulative PDL was calculated by adding the previous calculated PDL.

### Immunoblotting

Cells were lysed in RIPA buffer containing protease inhibitors (Sigma, Oakville, Canada). Equal amounts of proteins were resolved on 10 % SDS-PAGE and transferred to a nitrocellulose membrane (BioRad, CA, USA). Membranes were blocked in TBS containing 5 % non-fat dry milk and exposed overnight at 4 °C to rabbit-anti-BRCA1 (ab191042 and ab131360, Abcam, MA, USA) or mouse-anti-β-Actin (A5316, Sigma, Oakville, Canada). Membranes were washed in TBST (TBS-0.05 % Tween-20) and incubated with either anti-rabbit or anti-mouse peroxidase-conjugated secondary antibody for 1 h at room temperature. After several washes in TBST, the blots were developed using Immobilon Western HRP Substrate (Millipore, Etobicoke, Canada).

### Exosomes isolation and labeling

Exosomes were isolated from serum using Total Exosome Isolation kit according to the manufacturer’s protocol (Invitrogen, Burlington, Canada). Exosomes were labeled using the PKH26 dye following the manufacturer recommendations (Sigma, Oakville, Canada). Labeled exosomes were diluted in labeling stop solution (PBS/FBS) and pelleted by ultra-centrifugation for 80 min at 100,000 x g at 4 °C. The pellet was washed in Hank's Balanced Salt Solution (HBSS) with an ultra-centrifugation using the same parameters. The pelleted exosomes were re-suspended in HBSS and stored at −80 °C. 10 μg of labeled exosomes was added to ~5x10^3^*BRCA1*-KO Fibroblast, Control PX-458-transfected fibroblasts and HEK-293 cells cultured in 8-well chamber slides (VWR, Mont-Royal, Canada). Cells were washed, fixed for 10 min with Paraformaldehyde 4 %. Slides were mounted with coverslip in VECTASHIELD Mounting Medium with DAPI (Vector Laboratories, Burlington, Canada). Stained cells were visualized using an LSM780 confocal microscope (Zeiss, Toronto, Canada). Exosomes internalization was quantified using ImageJ software.

### Exosomes characterization

Morphological examination of isolated exosomes was done using transmission electron microscope (JEM-2010, Jeol Ltd., Tokyo, Japan). Briefly, 20 μl of exosomes were loaded on a copper grid and stained with 2 % phosphotungstic acid. Samples were dried by incubating them for 10 min under an electric incandescent lamp. Samples were examined under electron microscope and imaged using a Hitachi H-600 TEM operating at 60 kV. In Parallel, an aliquot of exosome samples was run on a Nanosight NS500 system (Nanosight Ltd., Amesbury, UK), and size distribution was analyzed using the NTA 1.3 software.

### In vivo tumor growth

Five-week-old female NOD-SCID mice (Jackson Laboratory) were used in compliance with McGill University Health Centre Animal Compliance Office (Protocol 2012–7280). Cells growing in log phase were harvested by trypsinization and washed twice with HBSS. Mice were injected subcutaneously with 2 million cells in 200 μl HBSS/Matrigel. Mice were euthanized one month post-injection. The resulting xenotransplants were photographed and processed as indicated below.

### Immunohistochemistry labelling procedures and histological analyses

Mice xenotransplants were collected, fixed in 10 % buffered formalin, embedded in paraffin, and stained with H&E (hematoxylin and eosin) according to standard protocols or processed for immunohistochemistry. Briefly, 5 μm tissue sections were dewaxed in xylene and rehydrated with distilled water. After antigen unmasking, and blocking of endogenous peroxidase (3 % hydrogen peroxide), the slides were incubated with primary antibodies (Additional file 3: Table S1). Labeling was performed using iView DAB Detection Kit (Ventana) on the Ventana automated immunostainer. Sections were counterstained lightly with Hematoxylin before mounting. Histological analyses were performed by a certified pathologist who was blinded to the type of cells from which the cancerous masses, which formed in mice, had been derived.

### Statistical analysis

Statistical analysis Statistical differences were analyzed using Student’s *t* test for unpaired samples. An ANOVA followed by the Dunnett test was used for multiple comparisons with one control group. The criterion for significance (p value) was set as mentioned in figures.

## Results

### *BRCA1* knocking-out in human fibroblasts

A human fibroblast cell line deficient for the oncosuppressor *BRCA1* was developed to study its susceptibility to the oncogenic potential of cancer patient serum (Additional file 1: Figure S1A). For this purpose, we used the CRISPR-Cas9 technology to knock-out *BRCA1* [31]. *BRCA1*-KO fibroblasts (i.e., fibroblasts transfected with a sgBRCA1-expressing vector) and control fibroblasts (empty vector transfected cells) were sorted based on the expression of the GFP reporter gene (Additional file 1: Figure S1B). *BRCA1* knockout was validated using the SURVEYOR nuclease assay (Additional file 2: Figure S2A). One of two guides used was efficient in knocking-out *BRCA1* in fibroblasts. As described in Materials and Methods, the Indel percentage was estimated at 33 %, which is in the range of the values obtained with this assay. Moreover, protein extracts were analyzed for BRCA1 expression. BRCA1 was not detected in *BRCA1*-KO fibroblasts, compared to empty vector-transfected cells (Additional file 2: Figure S2B).

### Cancer patient sera increased the proliferation of *BRCA1*-KO fibroblasts

**Fig. 1:**
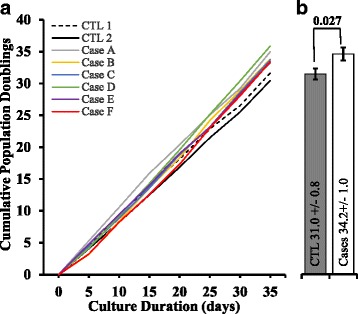
Cancer patient sera increased *BRCA1*-KO fibroblasts growth. Cells were cultured for 2 weeks in control human serum, or cancer patient sera. Cell growth was then analyzed by counting the number of viable cells at every passage (5 days duration for every passage). **a** Line graph shows the population doublings capability and (**b**) column graph represents cumulative population doublings at the end of the treatment periods. Data are mean ± SD of 2 control sera vs. 6 cancer patient sera. (*P* = 0.027)

To analyze the effect of cancer patient sera on the growth of *BRCA1*-deficient fibroblasts, equal amount of cells were plated and cultured with DMEM media supplemented with 10 % of cancer patients’ serum for 2 weeks. We used serum from two healthy control donors and 10 metastatic cancer patients (Table 1). At every passage, cell numbers were determined to estimate the population doubling levels (PDL) in each condition (Fig. 1a). Independently of the cancer serum used, the cumulative PDL was increased when compared to that of cells cultured in control human serum (range: 1.3 to 6.1 increase in population doubling; mean +/− SD: 3.2 +/− 1.4; *P* = 0.027) (Fig. 1b). These data suggest that cancer patient sera significantly enhanced the proliferation of *BRCA1*-deficient fibroblasts in vitro.

### Cancer patients’ sera transfer malignant traits to *BRCA1*-KO fibroblasts

**Fig. 2:**
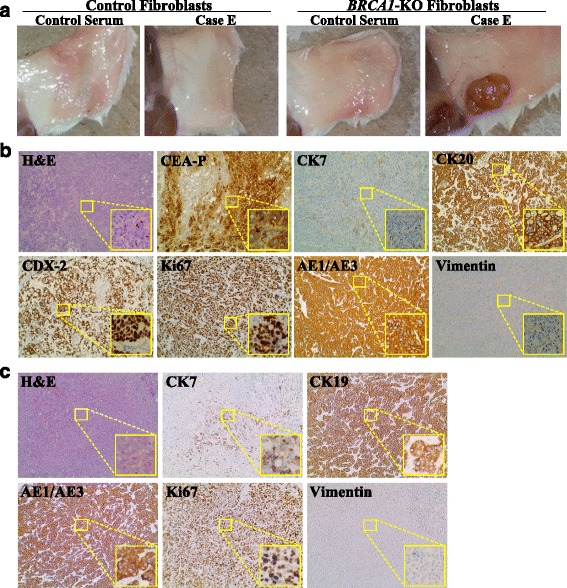
Cancer patient sera induced the transformation of *BRCA1*-KO fibroblasts. **a** NOD/SCID mice were injected with either control or *BRCA1*-KO fibroblasts, that were treated for 2 weeks with healthy individual (Control) or cancer patient sera. 4 weeks after cells transplantation, mice were euthanized and the tumors photographed. Representative pictures are shown. Tumor masses were only observed when mice were injected with *BRCA1*-KO fibroblasts treated with cancer patient sera. (*n* = 3 mice per group). (**b** and **c**) Formalin-fixed paraffin-embedded tumors generated following injection of *BRCA1*-KO fibroblasts treated with CRC-LM patient serum (**b**: Case E) or pancreatic cancer patient serum (**c**: Case J) were processed for H&E staining, or immunolabeled with antibodies against tumor specific markers

To determine whether cancer sera promote tumor formation in vivo, NOD/SCID mice were injected subcutaneously with *BRCA1*-KO fibroblasts exposed to healthy control or cancer patients’ sera. Cells were injected following 2 weeks of treatment. Mice were followed up for tumor growth, and they were euthanatized 3 to 4 weeks after cell inoculation due to the massive growth of cancerous masses, which had compromised the ability to move of the mice. Independently of the cancer serum used, all mice injected with cancer sera-treated *BRCA1*-KO fibroblasts developed visible tumors as early as one week following inoculation (Fig. 2a and Additional file 4: Figure S3). In contrast, none of the mice injected with control fibroblasts, treated with healthy control or cancer patient sera, developed tumors during the course of the experiments (4 weeks latency) (Fig. 2a).

**Fig. 3:**
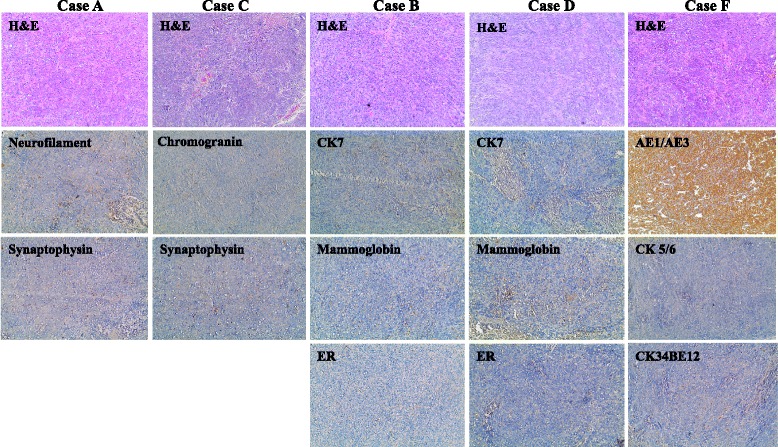
Tumors generated with cancer patient sera-treated *BRCA1*-KO did not all display differentiation characteristics. *BRCA1*-KO fibroblasts were treated with cancer patient sera for 2 weeks (Cases A, B, C, D, and F). Treated cells were injected into NOD/SCID mice that were followed for 4 weeks for tumors growth. Developing tumors were excised and photographed (See Additional file 4: Figure S3). Formalin-fixed paraffin-embedded xenotransplant samples were processed for H&E staining, or immunolabeled with antibodies against tumor specific markers

Histopathological analyses of excised tumors showed solid growth of tumor cells sheets with high proliferation index (over 85–90 % Ki67 positivity) (Fig. 2b, c and Additional file 5: Figure S4). We further characterized these tumors for differentiation patterns based on the primary tumor of the blood donors. The histology of tumors obtained with cells treated with sera derived from five different patients (Cases A, B, C, D and F) confirmed that they were poorly differentiated carcinomas (Fig. 3 and Additional file 4: Figure S3). Notably, vimentin staining of all the cancerous masses was negative indicating that cancer factors carried in the sera of all tested patients had integrated in the genome of the *BRCA1*-KO fibroblasts changing their fate permanently. Even more striking was the discovery that *BRCA*-KO fibroblasts treated with sera taken from four patients with colorectal cancer liver metastases (Cases E, G, H and I), and one patient with pancreatic cancer (Case J) displayed epithelial differentiating features typical of colorectal adenocarcinomas and pancreatic ductal adenocarcinoma, respectively (Table 3 and Fig. 2b, c and Additional file 5: Figure S4). The tumors generated with cells treated with cases E, G, H and I (colorectal cancer) were negative for CK7 but they all stained positive for CEA, CK20, CDX-2 and AE1/AE3, which are universal markers of colorectal cancer. The tumors generated with *BRCA*-KO fibroblasts treated with case J (pancreatic cancer), stained strongly positive for CK7/CK19 which is a typical feature of pancreatic adenocarcinoma differentiation. Interestingly, CK7 expression was not uniform in the cancer specimen but it was strongly expressed in the areas with pancreatic tumor morphology indicating an evolving differentiation from undifferentiated morphology to pancreatic cancer phenotype. Attempts to characterize immunohistochemically the other tumors failed to show any differentiating features resembling the tumors of blood donors as they all stained negatively for tumor-specific markers (Table 3 and Fig. 3).

**Table 3:** Summary of the immunohistochemistry analyses of the xenografts obtained with cancer patients’ serum-treated *BRCA1*-KO fibroblasts

Cases	Xenograft Phenotype	Differentiation Markers	
Case A	Poorly differentiated carcinoma	Synaptophysin Neurofilament	negative negative
Case B	Poorly differentiated carcinoma	Estrogen receptor Mammoglobin CK7	negative rare positivity rare positivity
Case C	Poorly differentiated carcinoma	Chromogranin Synaptophysin	negative rare positivity rare positivity
Case D	Poorly differentiated carcinoma	Estrogen receptor Mammoglobin CK7	negative negative rare positivity
Case F	Poorly differentiated carcinoma	CK5/6 CK34BE12 AE1/AE3	negative negative Strongly + ve
Case E	Differentiation toward intestinal adenocarcinoma	CEA CK7 CK20 CDX-2 AE1/AE3 Vimentin	positive negative positive positive positive negative
Case G	Differentiation toward intestinal adenocarcinoma	CEA CK7 CK20 CDX-2 AE1/AE3 Vimentin	positive negative positive positive positive negative
Case H	Differentiation toward intestinal adenocarcinoma	CEA CK7 CK20 CDX-2 AE1/AE3 Vimentin	positive negative positive positive positive negative
Case I	Differentiation toward intestinal adenocarcinoma	CEA CK7 CK20 CDX-2 AE1/AE3 Vimentin	positive negative positive positive positive negative
Case J	Differentiation toward typical pancreatic ductal adenocarcinoma	CK7 CK19 AE1/AE3 Vimentin	positive positive positive negative

### *BRCA1*-KO fibroblasts treated with healthy control sera failed to acquire any malignant transformation

To rule out the possibility that *BRCA1*-KO fibroblasts might have an innate tendency to turn malignant due to the impaired function of the *BRCA1* gene, *BRCA1*-KO fibroblasts were cultured with healthy patients’ serum for 2 weeks. Inoculation of these cells in NOD/SCID mice failed to form any mass even at longer latency period (12 weeks post-transplantation), confirming the known notion that a single oncosuppressor mutation is not enough to trigger cancerogenesis and activate the cascade of events that eventually lead to cancer transformation (Fig. 2a). Furthermore, the results of this test confirmed also that the putative factors responsible for the malignant transformation of the *BRCA1*-KO fibroblasts were exclusively present in the serum of patients with metastatic cancer and were absent in the serum of healthy patients.

### Malignant transformation of the *BRCA1*-KO fibroblasts is permanent and not transient

**Fig. 4:**
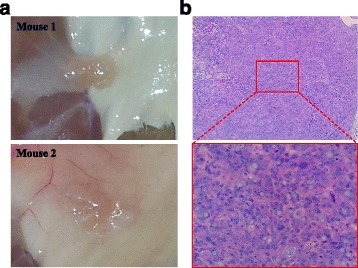
Cancer patient sera permanently transformed *BRCA1*-KO fibroblasts. **a** *BRCA1*-KO fibroblasts were treated with cancer patient serum for 2 weeks. Cells were then transferred to regular culture medium (10 % FBS-supplemented DMEM-F12 medium with 1 % antibiotics) for 2 weeks. Cells were injected subcutaneously into NOD/SCID mice. 4 weeks after cells transplantation, mice were euthanized and the tumors photographed. Representative pictures are shown. **b** Formalin-fixed paraffin-embedded xenotransplants samples were processed for H&E staining

Further, we wanted to test whether the malignant transformation of the *BRCA1*-KO fibroblasts was permanent and not secondary to a short-term and transient effect. Therefore, *BRCA1*-KO fibroblasts were treated with cancer serum for 2 weeks. Subsequently, they were exposed to DMEM-F12 media supplemented with 10 % FBS and 1 % Pen-Strep, for two more weeks to allow them to recover. Interestingly we found that cells had kept their tumorigenic potential and still formed tumors when they were injected into NOD/SCID mice, indicating thus that the transformation was indeed permanent and not transient (Fig. 4).

### *BRCA1*-KO fibroblasts demonstrate a significant increased uptake of cancer-derived exosomes

**Fig. 5:**
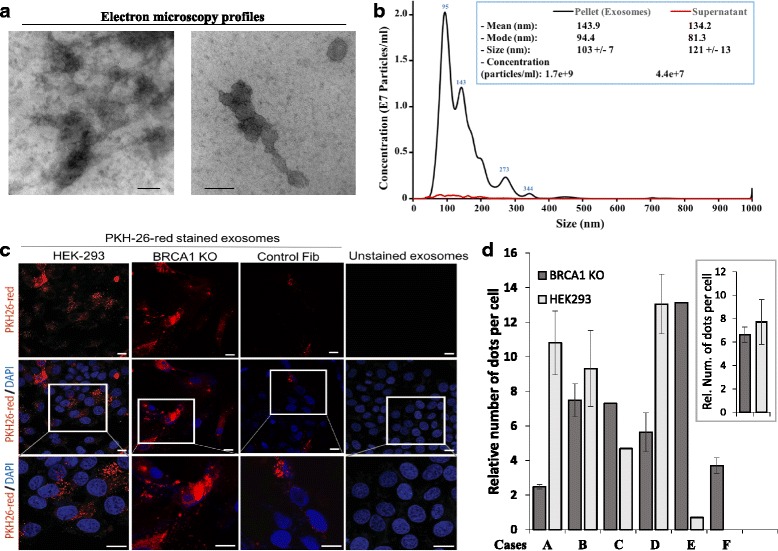
*BRCA1*-KO fibroblasts internalized exosomes more efficiently than control cells. **a** Exosomes were isolated as described under Methods. Representative micrographs of transmission electron microscopy on cancer patient sera exosome preparations. The image showed small vesicles of approximately 50–120 nm in diameter. Scale bars 100 nm. **b** NanoSight analysis of samples prepared as in (**a**). The size was centered around 94 nm in diameter. **c** Confocal microscopy monitoring of PKH-26-labeled exosome uptake in vitro into *BRCA1*-KO fibroblasts, control fibroblasts, and HEK293 cells. Note that exosomes internalized more in *BRCA1*-KO cells. They were dispersed in the cytoplasm and tended to form aggregates in the perinuclear regions. **d** The number of PKH-26-labeled exosomes (dots) was counted. Data are expressed as relative number (Rel. Num.) of exosomes per cell compared to that in control fibroblasts. In the insert, data are expressed as mean +/− SD (*n* = 6 exosome preparations; *P* = 0.032 for HEK293 cells compared to control fibroblasts, *P* = 0.028 for *BRCA1*-KO fibroblasts compared to control fibroblasts, and *P* = 0.35 for HEK293 cells compared to *BRCA1*-KO fibroblasts)

A number of recent evidences indicate that exosomes are involved in cancer invasion and metastasis [4, 8, 18, 32]. We had noticed in previous unpublished experiments that cancer exosomes had the tendency to accumulate in the cytoplasm of HEK293 cells in larger number than what was observed in normal cells. Following this observation, we hypothesized that perhaps one of the methods implemented by the oncosuppressor genes to protect the genome of the cells was to prevent the uptake of genetic material potentially harmful to the cell. As a consequence of that, any cell with a single oncosuppressor mutation would show a superior uptake of cancer exosomes. In order to test our hypothesis, exosomes were isolated from cancer patients’ sera. The size of the isolated particles was between 50 and 120 nm as visualized by electron microscopy (Fig. 5a), and Nanosight analyses (size = 103 +/− 7; Fig. 5b). This is in the range of the expected size for exosomes. The isolated exosomes were labeled with PKH-26 and added to *BRCA1*-KO and wild type fibroblasts, and HEK293 cell cultures to assess their internalization. Exosomes uptake was assessed by measuring the number of positive intracellular spots (Fig. 5c and d). As hypothesized, *BRCA1*-KO fibroblasts and HEK293 cells showed an uptake of cancer exosomes 3 to 13 times higher (6.6 +/− 0.6 times for *BRCA1*-KO fibroblasts and 7.7 +/− 2.0 for HEK293 cells) than that measured in control wild type fibroblasts. This suggests that one of the ways oncosuppressor genes might use to protect the integrity of the genome would be to act as gatekeepers at the membrane level and block the integration of dangerous genetic material.

## Discussion

In the present study, we confirmed that sera of patients with metastatic cancer contain tumorigenesis-signaling factors that, once delivered to recipient target cells, are capable to complete the cascade of events that eventually lead the cells to acquire malignant traits. In our previous research we had used the HEK293 cells as a model of “initiated” cell to demonstrate that the horizontal transfer of blood-circulating cancer factors is also applicable to human cells [26], confirming thus, the validity of the “genometastatic” theory in humans [23–25]. According to this theory, carcinogenetic steps such as initiation, promotion and progression might not represent events limited to the cells forming the primary tumor, but may actually be a process reproducible through cancer factors, shed by primary tumors and carried through the blood, to susceptible cells, located at metastatic sites [5, 7, 10–16]. In order to strengthen the validity of this alternative metastatic pathway in humans, we used fibroblasts, which are among the most represented cells in human body and central players at metastatic sites [33, 34]. In addition, these cells display a high level of plasticity [35]. We induced a *BRCA1* oncosuppressor mutation to reproduce an in vitro model that would be as close as possible to what it is encountered in real clinical scenarios. We hypothesized that not only immortal cells, as previously demonstrated [23–26], but also any human cell carrying a single oncosuppressor mutation might represent a target cell susceptible of malignant transformation if exposed to blood-circulating cancer factors. The observation that *BRCA1*-KO fibroblasts cultured in cancer patients’ sera displayed oncogenic properties such as increased proliferation potential and ability to form tumors following subcutaneous injection into NOD/SCID mice supports the belief that truly a horizontal transfer of cancer material might have been overlooked when trying to understand metastatic disease. Moreover, the discovery that *BRCA1*-KO fibroblasts change their fate completely and turn into colon cancer cells and pancreatic cancer cells, when exposed to serum derived from patients affected by metastatic colon and pancreatic cancer, strengthen the belief of the authors, that similarity doesn’t imply sameness and therefore metastatic cells might not necessarily be only cells detaching from primary tumors. In line with this concept, the molecular profiles of primary and metastatic lesions are not usually identical and therefore, at least theoretically, the possibility that metastases might not be deriving from the same cells is still open [36–39]. The data gathered in our study prove that any cell as long as it is “initiated” has the potentials to incorporate in its genome “signaling” factors, change its fate and acquire aberrant phenotypical traits identical or similar to the source of the signaling factors.

To our knowledge, this is the first study to demonstrate the transformation of human fibroblasts carrying a single oncosuppressor mutation (i.e., BRCA1) into colon cancer or pancreatic cancer cells after exposure to serum of patients with metastatic colon or metastatic pancreatic cancer. This result becomes even more striking and fascinating when considering that the recovery test indicated that the phenotypical modifications were indeed permanent and not related to a transient alteration of the genetic asset of the cells. In other words, a short exposure to cancer factors present in the serum seems to have been strong enough to overcome the repair mechanisms of the treated cells. The main implication of this stable modification of the genome of human cells is that at least in vitro, metastatic transformation, through horizontal transfer, is not a theory anymore but a fascinating reality. The scientific soundness of this data is corroborated by the evidence brought about in our experiments that the malignant transformation would be unlikely to be secondary to an innate instability of the fibroblasts caused by the *BRCA1* mutation. In fact, the exposure of the *BRCA1*-KO cells to healthy serum never made them susceptible of malignant transformation, confirming the known notion that a single mutation is not able to trigger the cascade of events that leads to tumorigenesis [40].

In our study, we documented, for the first time, a significant increased uptake of cancer-derived exosomes by *BRCA1*-KO fibroblasts and HEK293 cells when compared to wild type fibroblasts. This finding noted only in cells carrying a single oncosuppressor mutation suggests a potential role that oncosuppressor genes might have in exosomes trafficking. Perhaps, they might protect the integrity of the cellular genome not only by repairing DNA damages and controlling cell cycle checkpoints [27, 41], but also by blocking the uptake of extracellular mutating material and thus preventing their integration in the genome with subsequent DNA alterations. Although we reported an increased uptake of cancer derived exosomes in oncosuppressor mutated cells, their putative role in the documented malignant transformation of the cells yet has to be determined as well as the true nature of all the factors involved in the transfer of malignant traits. While cancer cells-derived circulating factors (i.e., DNA, mRNA, miRNA, proteins) were detected in the blood of cancer patients, and their regulatory role in cancer progression and development were reported in many studies [16, 22, 32, 42, 43] still their respective roles have to be fully defined. If on one hand, it has been reported that exosomes originating from the primary tumor paves the way for organ-specific metastasis by preparing a niche for the engraftment of circulating cancer cells, [5–7, 9], on the other hand, the results of our study might offer a different prospective, which do not exclude, but might integrate the conventional view on metastasis.

The inability of the *BRCA1*-KO fibroblasts to fully differentiate in all cancer phenotypes is a limitation of our study. In our view, this failure is not perceived necessarily as a weakness of the theory but it is perceived as awareness that the genometastatic process is not understood in its entirety. When we exposed HEK293 to cancer patients’ sera the cells turned only into poorly differentiated carcinomas regardless of the type of cancer studied [26]. The exposure of *BRCA1*-KO fibroblasts has added value to the genometastatic theory since we have observed a full differentiation into at least two cancer cell lineages (colon and pancreas). We are planning to repeat the experiments with different cells and different single oncosuppressor mutations to see if the horizontal transformation targets different types of cells in different organs and at different stages of their physiological differentiation according to the type of cancer.

## Conclusion

The data presented in this study support the hypothesis that any human cells carrying a single oncosuppressor mutation is capable of integrating cancer factors carried in the blood of cancer patients and undergo complete malignant transformation. The evidence shown in our experiments that uptake of cancer-derived exosomes is significantly increased in these cells suggests a possible role that oncosuppressor genes might have in exosomes trafficking.

The reported findings support the notion of the possible role of a non-classical pathway to explain cancer traits exchange between malignant and non-malignant cells that may have implications during cancer progression and metastasis. Based on these results, the hypothesis that dissemination and migration of cancer cells from primary tumors might not be the only mechanism to explain metastases seems rational and merits further study.

## Additional files

Additional file 1: Figure S1. Methodology design followed to knock-out *BRCA1* in fibroblasts. pX-458 plasmid was used as a vector for CRISPR-Cas9 system for *BRCA1* knocking down. Human fibroblasts were transfected using lipofectamine 3000 (A). Transfected fibroblasts with sgBRCA1-pX458 or empty pX-458 vectors were sorted using a FACSAria cell sorter based on their GFP positivity. Only GFP positive cells were obtained and expanded in culture (B). (i) Naïve fibroblasts were gated as negative fraction (GFP negative fibroblasts). (ii) Fraction of cells sorted as control fibroblasts (empty pX-458 vector-transfected cells). (iii) Fraction of cells sorted as sgBRCA1-pX458 transfected fibroblasts (*BRCA1*-KO). Transfection efficiency showed percentage of 4–6 %. Fibroblasts were treated with cancer patients’ serum or healthy individual serum. Treated cells were analyzed for their in vitro proliferation or injected into NOD/SCID mice for tumor growth potential. (PPT 524 kb) Additional file 2: Figure S2. Validation of *BRCA1* Knockout in human fibroblasts. (A) Surveyor nuclease assay was performed as described under Materials and Methods. DNA was extracted from sorted fibroblasts. After amplification, DNA was denatured, reannealed and subjected to endonuclease digestion. Digestion products were run on 1 % agarose gel. Only with DNA extracted from sgBRCA1-transfected fibroblasts, we detected 3 bands: the full-length PCR product (i) and the digestion products (ii and iii). Note that the cumulative size of bands ii and iii equals the size of band i. (B) Western blot analysis of proteins extracted from control fibroblasts (empty vector transfected) and *BRCA1*-KO fibroblasts. Note that BRCA1 signal is absent in BRCA1-KO fibroblasts. (*) points to the BRCA1 signal. (PPT 265 kb) Additional file 3: Table S1. List of antibodies used in this study. Table S2. BRCA1 guide sequence as designed by the CRISPR Design Tool. Table S3. Analyses of off-target sequences of the BRCA1 guide. (DOC 58 kb) Additional file 4: Figure S3. Cancer patient sera induced the transformation of *BRCA1*-KO fibroblasts. *BRCA1*-KO fibroblasts were treated with cancer patient sera for 2 weeks (6 Cases). Treated cells were injected into NOD/SCID mice that were followed for 4 weeks for tumors growth. Developing tumors were excised and photographed. (PPT 189 kb) Additional file 5: Figure S4. Cancer patient sera changed the fate of *BRCA1*-KO fibroblasts. *BRCA1*-KO fibroblasts were treated with CRC-LM patient sera for 2 weeks (Cases G, H and I). Treated cells were injected into NOD/SCID mice that were followed for 4 weeks for tumors growth. Generated tumors were processed for H&E staining, or immunolabeled with antibodies against tumor specific markers. (PPT 36551 kb)

## Abbreviations

ANOVA: analysis of variance
*BRCA1*: breast cancer susceptibility gene 1
BSA: bovine serum albumin
DAB: diaminobenzidine
FBS: fetal bovine serum
H&E: hematoxylin and eosin
HEK293: human embryonic kidney cells
KO: knockout
PDL: Population doubling level
