# Supplementary material for: Transfer of malignant trait to BRCA1 deficient human fibroblasts following exposure to serum of cancer patients
Source: J Exp Clin Cancer Res. 2016 May 14;35:80. doi: 10.1186/s13046-016-0360-9 (PMC4868000; doi:10.1186/s13046-016-0360-9)
Supplement: Additional file 1: Figure S1. — Methodology design followed to knock-out BRCA1 in fibroblasts. pX-458 plasmid was used as a vector for CRISPR-Cas9 system for BRCA1 knocking down. Human fibroblasts were transfected using lipofectamine 3000 (A). Transfected fibroblasts with sgBRCA1-pX458 or empty pX-458 vectors were sorted using a FACSAria cell sorter based on their GFP positivity. Only GFP positive cells were obtained and expanded in culture (B). (i) Naïve fibroblasts were gated as negative fraction (GFP negative fibroblasts). (ii) Fraction of cells sorted as control fibroblasts (empty pX-458 vector-transfected cells). (iii) Fraction of cells sorted as sgBRCA1-pX458 transfected fibroblasts (BRCA1-KO). Transfection efficiency showed percentage of 4–6 %. Fibroblasts were treated with cancer patients’ serum or healthy individual serum. Treated cells were analyzed for their in vitro proliferation or injected into NOD/SCID mice for tumor growth potential. (PPT 524 kb) [file 13046_2016_360_MOESM1_ESM.ppt]

## Slide 1
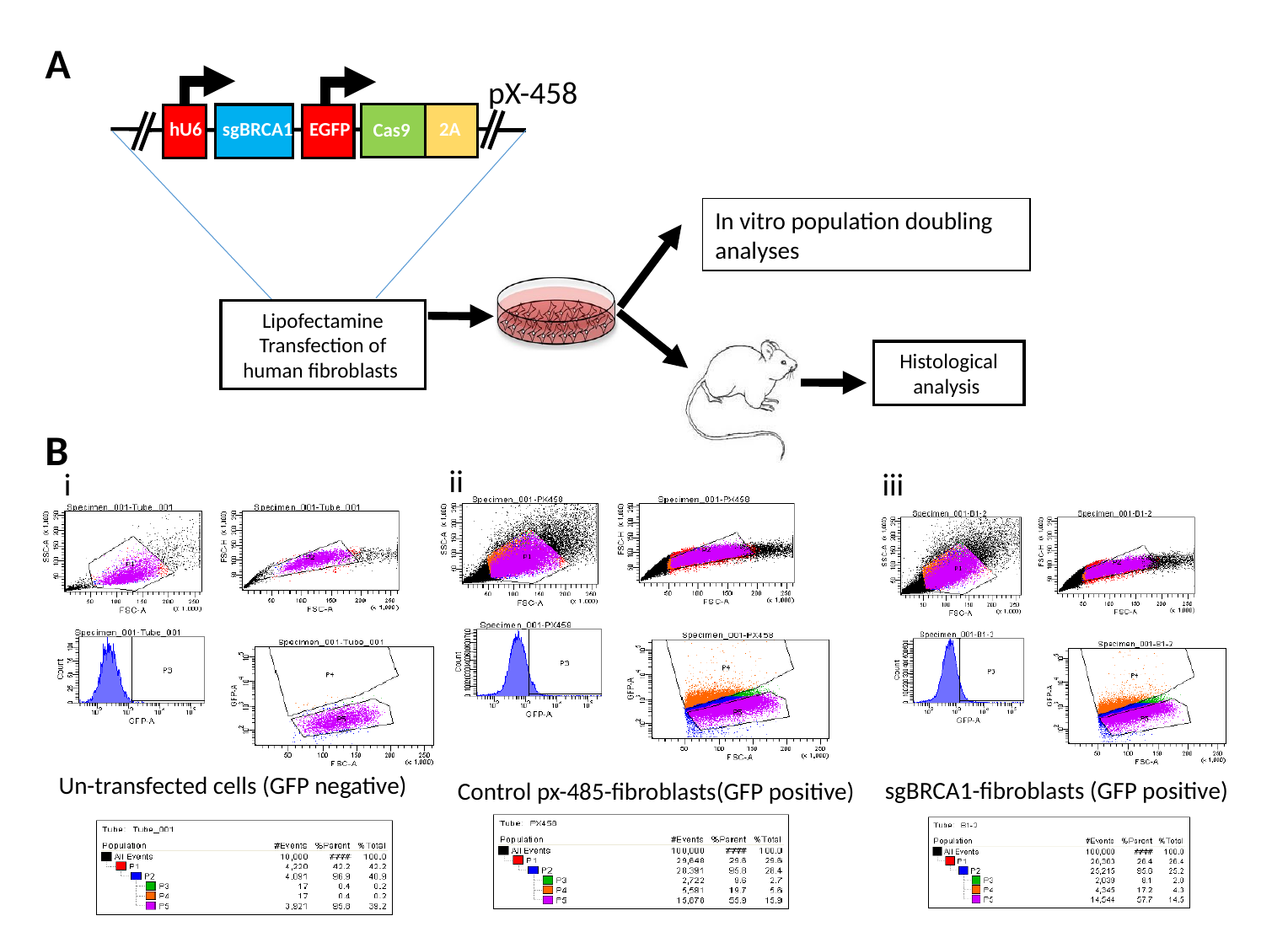

A
pX-458
sgBRCA1
2A
hU6
EGFP
Cas9
Lipofectamine Transfection of human fibroblasts
Histological analysis
In vitro population doubling analyses
B
ii
i
iii
Un-transfected cells (GFP negative)
sgBRCA1-fibroblasts (GFP positive)
Control px-485-fibroblasts(GFP positive)
