# Supplementary material for: Transfer of malignant trait to BRCA1 deficient human fibroblasts following exposure to serum of cancer patients
Source: J Exp Clin Cancer Res. 2016 May 14;35:80. doi: 10.1186/s13046-016-0360-9 (PMC4868000; doi:10.1186/s13046-016-0360-9)
Supplement: Additional file 2: Figure S2. — Validation of BRCA1 Knockout in human fibroblasts. (A) Surveyor nuclease assay was performed as described under Materials and Methods. DNA was extracted from sorted fibroblasts. After amplification, DNA was denatured, reannealed and subjected to endonuclease digestion. Digestion products were run on 1 % agarose gel. Only with DNA extracted from sgBRCA1-transfected fibroblasts, we detected 3 bands: the full-length PCR product (i) and the digestion products (ii and iii). Note that the cumulative size of bands ii and iii equals the size of band i. (B) Western blot analysis of proteins extracted from control fibroblasts (empty vector transfected) and BRCA1-KO fibroblasts. Note that BRCA1 signal is absent in BRCA1-KO fibroblasts. (*) points to the BRCA1 signal. (PPT 265 kb) [file 13046_2016_360_MOESM2_ESM.ppt]

## Slide 1
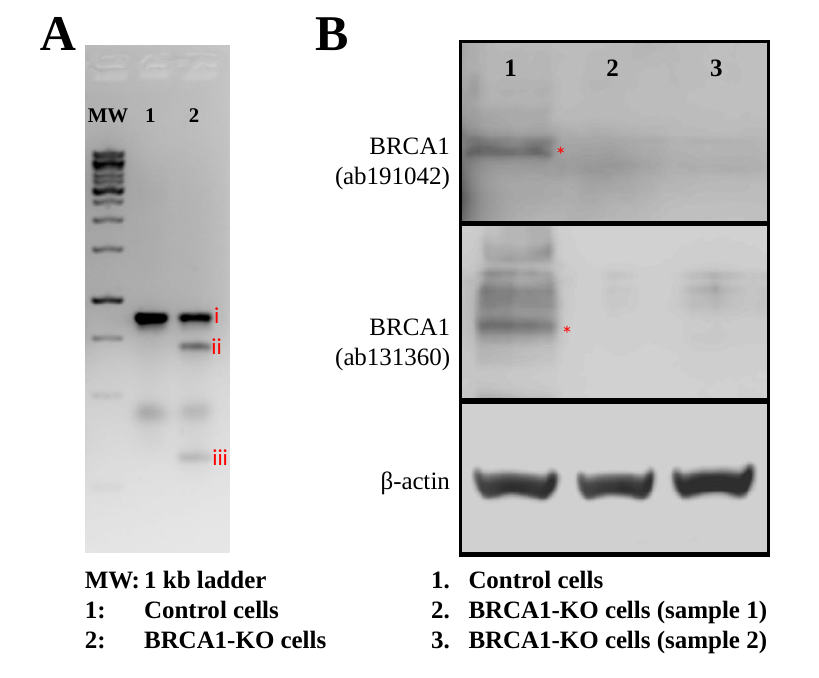

A
B
1
2
3
*
*
MW
1
2
 BRCA1
(ab191042)
i
 BRCA1
(ab131360)
ii
iii
 β-actin
MW:	1 kb ladder
1:	Control cells
2:	BRCA1-KO cells
Control cells
BRCA1-KO cells (sample 1)
BRCA1-KO cells (sample 2)
