# Supplementary material for: Transfer of malignant trait to BRCA1 deficient human fibroblasts following exposure to serum of cancer patients
Source: J Exp Clin Cancer Res. 2016 May 14;35:80. doi: 10.1186/s13046-016-0360-9 (PMC4868000; doi:10.1186/s13046-016-0360-9)
Supplement: Additional file 3: Table S1. — List of antibodies used in this study. Table S2. BRCA1 guide sequence as designed by the CRISPR Design Tool. Table S3. Analyses of off-target sequences of the BRCA1 guide. (DOC 58 kb) [file 13046_2016_360_MOESM3_ESM.doc]

**Supplementary Table1**. List of antibodies used in this study.

| Antibodies ID | Species | Manufacturer |
| --- | --- | --- |
| Cytokeratin 7 | Mouse Monoclonal | DAKO (Denmark) |
| Cytokeratin 20 | Mouse Monoclonal | DAKO (Denmark) |
| CEA-P | Rabbit Polyclonal | DAKO (Denmark) |
| CDX-2 | Rabbit Monoclonal | Cell MARQUE (USA) |
| Ki67 | Rabbit Monoclonal | Ventana (USA) |
| AE1/AE3 | Mouse Monoclonal | DAKO (Denmark) |
| Vimentin | Mouse Monoclonal | Ventana (USA) |
| Synaptophysin | Rabbit Monoclonal | Ventana (USA) |
| Neurofilament | Mouse Monoclonal | DAKO (Denmark) |
| Estrogen Receptor (ER) | Rabbit Monoclonal | Ventana (USA) |
| Mammoglobin | Mouse and rabbit cocktail | Cell MARQUE (USA) |
| Chromogranin | Mouse Monoclonal | Ventana (USA) |
| CK5/6 | Mouse Monoclonal | Cell MARQUE (USA) |
| CK34BE12 | Mouse Monoclonal | DAKO (Denmark) |
| Cytokeratin 19 | Mouse Monoclonal | DAKO (Denmark) |

**Supplementary Table 2. BRCA1 guide sequence as designed by the CRISPR Design Tool.**

|  | Score | Sequences | PAM | Off target | In gene |
| --- | --- | --- | --- | --- | --- |
| BRCA1 Guide | 97 | TGGTCACACTTTGTGGAGAC | AGG | 40 | 15 |

Note that this guide is highly ranked (score of 97).

**Supplementary Table 3. Analyses of off-target sequences of the BRCA1 guide.**

| sequence | mismatches | UCSC gene | Transcripts |
| --- | --- | --- | --- |
| TGGGCTGCCTTTGTGGAGACAGG | 4MMs [4:6:7:8] | NM_016128 | COPG1 |
| TGCTCAAACTGGGTGGAGACTGG | 4MMs [3:7:11:12] | NM_001145101 | BTBD18 |
| TGCTCAAACCTTGTGGACACAGG | 4MMs [3:7:10:18] | NM_004947 | DOCK3 |
| TGATCACAGTATCTGGAGACAGG | 4MMs [3:9:11:13] | NM_001083961 | WDR62 |
| TGGACACAATCTCTGGAGACAGG | 4MMs [4:9:11:13] | NM_203422 | LRRN4CL |
| AGGGCACACTGTGTGAAGACTGG | 4MMs [1:4:11:16] | NM_001029869 | PLAC8L1 |
| TGGTCACTTTCTGTGCAGACCAG | 4MMs [8:9:11:16] | NM_003715 | USO1 |
| TGCTCACACTGAGGGGAGACAGG | 4MMs [3:11:12:14] | NM_032251 | CCDC88B |
| TGGCCACACTGAGAGGAGACAGG | 4MMs [4:11:12:14] | NM_002615 | SERPINF1 |
| TGTTCACACTTTGTAGATATTAG | 4MMs [3:15:18:20] | NM_017784 | OSBPL10 |
| TGTTCACACTTTTTTGAAACAGG | 4MMs [3:13:15:18] | NM_198892 | BMP2K |

- To reduce the abundance of similar targeted sequence, we used a web-based prediction algorithm tool (31). We used the highly ranked guide sequence (score 97) with the least exonic off-target sites. Of the 40 off-target sequences that arisen, 11 were in exons. Note that none of these genes is involved in tumorigenesis.
