# Supplementary figures and images for: Transfer of malignant trait to BRCA1 deficient human fibroblasts following exposure to serum of cancer patients
Source: J Exp Clin Cancer Res. 2016 May 14;35:80. doi: 10.1186/s13046-016-0360-9 (PMC4868000; doi:10.1186/s13046-016-0360-9)

## Slide 1
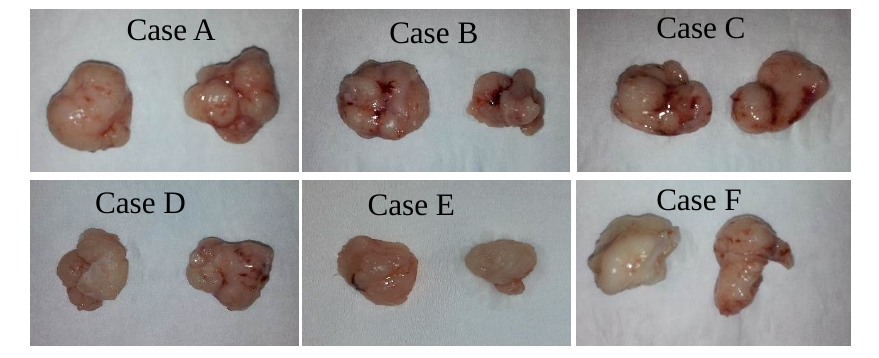

Case C
Case A
Case B
Case F
Case D
Case E

Supplement: Additional file 4: Figure S3. — Cancer patient sera induced the transformation of BRCA1-KO fibroblasts. BRCA1-KO fibroblasts were treated with cancer patient sera for 2 weeks (6 Cases). Treated cells were injected into NOD/SCID mice that were followed for 4 weeks for tumors growth. Developing tumors were excised and photographed. (PPT 189 kb) [file 13046_2016_360_MOESM4_ESM.ppt]

## Slide 1
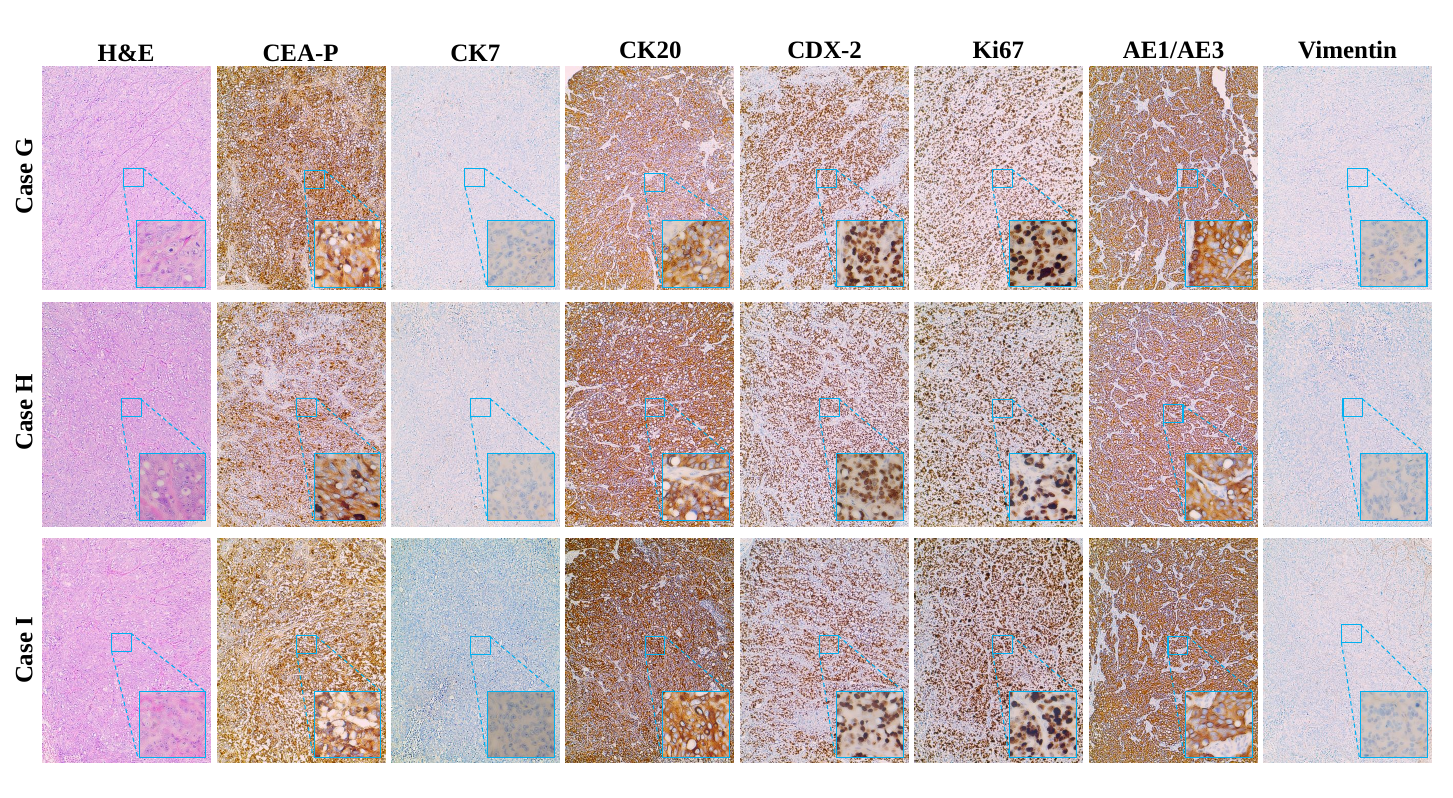

CK20
CDX-2
Ki67
AE1/AE3
Vimentin
H&E
CK7
CEA-P
Case G
Case H
Case I

Supplement: Additional file 5: Figure S4. — Cancer patient sera changed the fate of BRCA1-KO fibroblasts. BRCA1-KO fibroblasts were treated with CRC-LM patient sera for 2 weeks (Cases G, H and I). Treated cells were injected into NOD/SCID mice that were followed for 4 weeks for tumors growth. Generated tumors were processed for H&E staining, or immunolabeled with antibodies against tumor specific markers. (PPT 36551 kb) [file 13046_2016_360_MOESM5_ESM.ppt]
